# Rice residue promotes mobilisation and plant acquisition of soil phosphorus under wheat (*Triticum aestivum*)-rice (*Oryza sativa*) cropping sequence in a semi-arid Inceptisol

**DOI:** 10.1038/s41598-023-44620-7

**Published:** 2023-10-16

**Authors:** Avijit Ghosh, Dipak Ranjan Biswas, Ranjan Bhattacharyya, Shrila Das, Tapas K. Das, Khajanchi Lal, Supradip Saha, Pushpendra Koli, Rongrong Shi, Khurshid Alam, Yonglin Ren

**Affiliations:** 1 ICAR –Indian Agricultural Research Institute, New Delhi, 110 012 India; 2 College of Science, Health, Engineering and Education, Murdoch University, Perth, WA 6150 Australia; 3 ICAR –Indian Grassland and Fodder Research Institute, Jhansi, 284 003 India

**Keywords:** Element cycles, Agroecology

## Abstract

Disposal of significant tonnages of rice straw is expensive, but using it to mobilise phosphorus (P) from inorganically fixed pools in the soil may add value. This study was carried out to determine whether the use of rice straw mixed with phosphorus-solubilizing microbes could solubilize a sizable portion fixed soil P and affect P transformation, silicon (Si) concentration, organic acid concentrations, and enzyme activity to increase plant growth. Depending on the soil temperature, the application of rice straw at 12 Mg ha^−1^ with phosphorus-solubilizing microbes could solubilize 3.4–3.6% of inorganic P*,* and minimised the hysteresis impact by 6–8%. At plant maturity, application of rice straw at 12 Mg ha^−1^ with phosphorus-solubilizing microbes and 75% of recommended P application raised the activity of dehydrogenase, alkaline phosphatase activity, cellulase, and peroxidase by 77, 65, 87, and 82% in soil, respectively. It also boosted Si concentration in the soil by 58%. Wheat grain yield was 40% and 18% higher under rice straw at 12 Mg ha^−1^ with phosphorus-solubilizing microbes with 75% of recommended P application than under no and 100% P application, respectively. Rice grain yield also increased significantly with the same treatment. Additionally, it increased root volume, length, and P uptake by 2.38, 1.74 and 1.62-times above control for wheat and 1.98, 1.67, and 2.06-times above control for rice, respectively. According to path analysis, P solubilisation by Si and organic acids considerably increased (18–32%) P availability in the rhizosphere. Therefore, cultivators could be advised to use rice straw at 12 Mg ha^−1^ with phosphorus-solubilizing microbes with 75% P of mineral P fertiliser to save 25% P fertiliser without reducing wheat and rice yield.

## Introduction

Burning field crop waste has become a yearly occurrence in the heavily populated rural regions of Southeast Asia, China, and India^1^. Farmers have been compelled by the constant need to boost agricultural output to utilise unsustainable and agroecosystem-damaging methods. This is especially true in north-western India, where agricultural residue burning is widespread. Although the National Policy for Management of Crop Residues was adopted, routine burning of crop wastes persists^2^. In India, field burning accounts for over 16% of all agricultural waste, with rice straw making up 60% of that trash, to reduce the burden of trash disposal^3^. This is a significant source of air-borne particles and carbon emissions, affecting air quality and, ultimately, global climate. However, if managed properly, crop residues can be used to maintain soil organic matter, a crucial factor in determining the quality of the soil, and, when mineralized, recycle nutrients^4,5^.

For the wheat crop, P is a crucial macronutrient^6^. P limitation in wheat crops occurs mostly because of formation of the inorganic components, such as iron (Fe) phosphate, aluminium (Al) phosphate, and calcium (Ca) phosphate complexes^6^ P accumulates in the soil when P input from various sources is higher than P depletion due to crop removal. The surplus P from fertiliser tends to accumulate as inaccessible iron phosphate, aluminium phosphate, and calcium phosphate complexes in semi-arid Inceptisols of India^7,8^. Even while inorganic and organic P are abundant throughout most croplands, they are immobile and largely inaccessible to plants. There is a need to increase soil P availability for cropped plants.

The sorption–desorption properties of the soil regulate the availability of applied or solubilised P. Organic residues such as those from rice straw increase P availability in soils by preventing the formation of exposed hydroxyls on the surface of Fe and Al minerals^6^, as well as the competition between organic acids and phosphate ions for adsorbing sites^9^. In addition to this, upon decomposition of rice straw, silicate ions ((SiO_4_)^4^) are released. Silicate and phosphate ions compete with one another in solution for adsorbing sites on Al or Fe complexes, leading to the formation of Fe/Al–silicate and liberation of phosphate^10^. According to Nwoke et al.^11^ the impact of organic residues on P availability varied and was influenced by both the composition of the organics and soil properties. Relative to no residue, corn stover (residue) enhanced P in soil solution; however, the amplitude of the increase was higher in soils with low P sorption capacity than in soils with high P sorption capacity. Organic wastes can also solubilise native soil P to its available form^11,12^. The impact of application crop residues on soil P transformation and its role in reducing the hysteresis impact of P by controlling adsorption–desorption phenomena requires greater clarification prior to its wide scale adaption. This is a compelling issue because the contribution of rice residues to the P nutrition of the wheat crop in the Indo-Gangetic plain of South Asia could be significant.

The ideal rice straw application rate for P solubilisation is complex, depending on soil type, climate, and agronomic factors. An extremely high rice straw application rate might, because of the priming effect, immobilise soil nutrients^13^. On the other hand, a little amount of rice straw might not be adequate to release enough Si and organic acids to significantly mobilise fixed P^14^. It may be possible to solubilize the adsorbed or fixed inorganic P and diminish rice straw combustion by using rice straw combined with P solubilizing microorganisms. Addition of phosphorus-solubilizing microbes would hasten rice straw breakdown as well as soil acidification to solubilize resistant P compounds in soils. By reducing P deficiency, promoting plant absorption of P, and increasing Si availability to plants in the soil, the application of rice straw could improve crop yield^15,16^.

Further research is required to determine the unique mechanisms and processes occurring in the soil, the quantity of rice straw, and their consequences on the growth and production of crops. Only then can the ideal amount of rice straw for P solubilisation without reducing yield be recommended. A very high amount of straw application could risk crop yield by immobilization of significant amount of essential nutrients, such as, nitrogen, phosphorus, sulphur; whereas, a very low amount of straw application might not serve the purpose of supplying adequate quantity of Si and organic acids to solubilise recalcitrant P^13^. There is a lack of knowledge on how applying rice straw with phosphorus-solubilizing microbes impacts P mobilisation, the concentration of organic acids, the activity of soil enzymes, and the root architecture of plants. We postulated that using rice straw with phosphorus-solubilizing microbes would speed up the solubilisation of inorganic fixed P by lowering the hysteresis effect and modifying the soil environment by increasing the level of organic acids and competitive ions like silicate. The objective of this study was to understand the effects of crop residue and P management strategies on crop yield, transformation, solubilisation, and mineralization pattern of P in addition to P availability in soil–plant systems.

Consequently, this study’s design included two experiments. The first experiment was carried out under laboratory conditions to determine amounts of rice straw with phosphorus-solubilizing microbes should be applied to solubilize fixed P in soil and desorb the adsorbed P by minimising the hysteresis impact. The best combinations determined in experiment 1 were used in experiment 2 to assess their effects on wheat (*Triticum aestivum*) and rice (*Oryza sativa*) growth, soil P transformation, Si concentration, organic acid concentration, and enzyme activity.

## Results

### P mineralization and transformation

The use of rice straw with phosphorus-solubilizing microbes improved P mobilisation significantly (Table 1). T6 mobilised the most P at 25 °C and 35 °C. For T6, cumulative P mobilisation at 90 days after incubation (DAI) was 58%, 35%, and 20% greater at 25 °C than those of T2, T4, and T8. Gross P mobilisation at 90 DAI was 49, 30, and 12% higher for T6 at 35 °C than those of T2, T4, and T8, correspondingly (Table 1). Despite having greater rice straw, T6 had considerably higher cumulative P mobilisation than did T8 at 25 °C and 35 °C.

**Table 1:** Impact of rice straw and phosphorus-solubilizing microbes application on cumulative P mineralization (Pt, mg kg^−1^), organic P mineralization (Po; mg kg^−1^), rate of solubilisation of inorganic P (k1; day^−1^), and rate of solubilisation of organic P (k2; day^−1^) at 25 °C and 35 °C temperature in a semi-arid Inceptisol.

Temperature	25 °C				35 °C			
Treatments	Pt	Po	k1	k2	Pt	Po	k1	k2
T0	13.49 ± 2.04f	13.26 ± 1.06d	0.173 ± 0.04d	0.001 ± 0.01f	13.63 ± 2.29 f	13.07 ± 1.98e	0.313 ± 0.02e	0.001 ± 0.01 h
T1	22.05 ± 2.16e	14.30 ± 1.11cd	0.383 ± 0.05b	0.087 ± 0.01e	29.50 ± 2.76e	21.01 ± 2.06d	0.564 ± 0.04c	0.106 ± 0.08 g
T2	28.47 ± 2.01d	14.65 ± 1.23cd	0.700 ± 0.06a	0.092 ± 0.01e	36.01 ± 2.91d	26.84 ± 2.16b	0.900 ± 0.07a	0.141 ± 0.04e
T3	23.69 ± 2.14e	14.94 ± 1.76cd	0.383 ± 0.02b	0.099 ± 0.01e	31.24 ± 2.83de	21.72 ± 2.42d	0.564 ± 0.04c	0.1170.02 ± f
T4	33.39 ± 2.64c	16.59 ± 1.60b	0.700 ± 0.06a	0.129 ± 0.02d	41.24 ± 3.43c	28.97 ± 2.53b	0.900 ± 0.07a	0.173 ± 0.05c
T5	27.59 ± 2.11d	15.42 ± 1.29bc	0.383 ± 0.02b	0.124 ± 0.02d	35.41 ± 3.24d	23.09 ± 2.09c	0.564 ± 0.04c	0.159 ± 0.06d
T6	45.09 ± 3.42a	18.02 ± 1.30a	0.700 ± 0.06a	0.205 ± 0.03a	53.76 ± 3.59a	33.08 ± 2.67a	0.900 ± 0.07a	0.301 ± 0.106a
T7	25.13 ± 2.10de	13.24 ± 1.24d	0.383 ± 0.02b	0.117 ± 0.02d	33.52 ± 2.93cd	22.98 ± 2.14cd	0.540 ± 0.04c	0.155 ± 0.06d
T8	37.70 ± 2.73b	11.49 ± 1.64e	0.700 ± 0.06a	0.184 ± 0.02b	48.07 ± 3.46b	32.75 ± 3.37a	0.826 ± 0.05b	0.289 ± 0.07b
T9	24.19 ± 2.67de	14.98 ± 1.55cd	0.276 ± 0.01c	0.167 ± 0.02c	38.86 ± 2.95cd	23.12 ± 2.11c	0.480 ± 0.04d	0.175 ± 0.06c

Means with a similar lower-case letter within a column are not significantly different according to Tukey’s HSD test (*p* < 0.05).

When rice straw was supplied alone at various doses, P mobilisation increased. At all temperatures, P mobilisation increased when phosphorus-solubilizing microbes were included with rice straw. The rate of organic P mineralisation was similar under T2, T4, and T6, and was 4- and 2.5-fold greater than under T0 and T9. T1, T3, and T5 exhibited identical rates of organic P mineralisation, which were 2.21 and 1.39 times greater than T0 and T9, correspondingly, at 25 °C. At 35 °C, similar outcomes were observed. At 25 °C, the rate of inorganic P solubilisation was 2.2, 1.6, and 1.1-fold greater under T6 than under T2, T4, and T8. However, at 35 °C, the rate of inorganic P solubilisation under T6 was 2.1, 1.7, and 1.0 times higher than under T2, T4, and T8 (Table 1).

**Figure 1:**
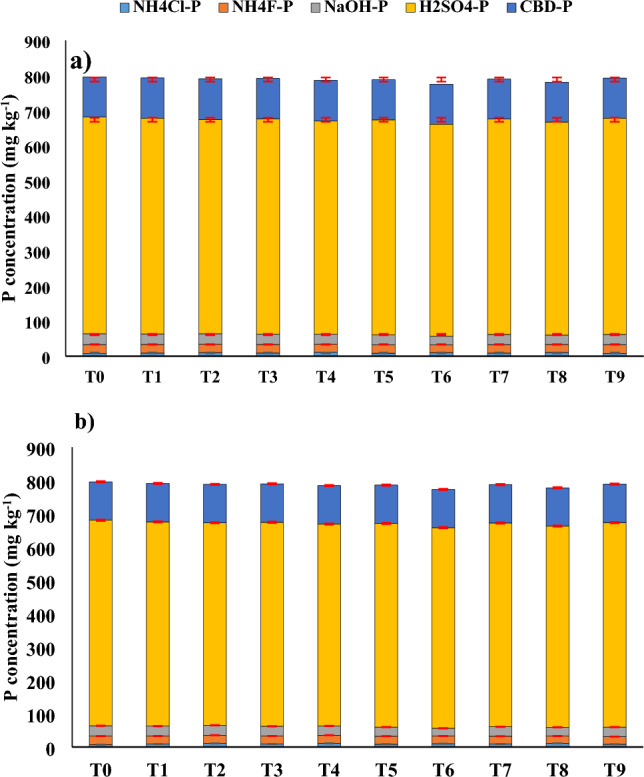
Changes in ammonium chloride extractable P (NH_4_Cl-P), ammonium fluoride extractable P (NH_4_F-P), sodium hydroxide extractable P (NaOH-P); citrate-bicarbonate extractable P (CBD-P), and sulfuric acid extractable P (H_2_SO_4_-P) as impacted by varying doses of rice straw application and phosphorus solubilising microbes’ application at (**a**) 25 °C and (**b**) 35 °C in a semi-arid subtropical Inceptisol. Error bars indicate confidence interval at 95% confidence level.

Usage of rice straw alone aided to transform inorganic soil P considerably in all cases. T4, T6, and T8 enhanced NH_4_Cl-P by 52%, 33%, and 40%, respectively at 25 °C. Over T0, T6 and T8 mobilised 13.4 and 10.3% of NH_4_F-P, 17.7% and 12.7% of NaOH-P, and 2.28% and 1.74% of H_2_SO_4_-P (Fig. 1). The citrate-bicarbonate extractable P, on the other hand, did not differ significantly. Nonetheless, net inorganic P solubilisation was 27.1 and 26.2 mg kg^−1^ under T6 and T8, respectively. Organic P mineralization was 56% greater under T6 than under T8. T4, T6, and T8 increased NH_4_Cl-P by 50%, 35%, and 54%, respectively, at 35 °C. Compared to T0, T6 and T8 mobilised 8% and 21% of NH_4_F-P, 25% and 15% of NaOH-P, and 2.43% and 2.11% of H_2_SO_4_-P (Fig. 1). Net inorganic P solubilisation was 20.7 and 15.3 mg kg^−1^ under T6 and T8, respectively. Organic P mineralization for T6 was comparable to T8. At 35 °C, cumulative P mobilisation was higher than at 25 °C (Table 1).

### P hysteresis and Si availability

The combination of rice straw and phosphorus-solubilizing microbes had a significant effect on the adsorption isotherms, whereas rice straw or phosphorus-solubilizing microbes separately did not. T6 and T8 greatly minimised hysteresis and adsorption parameters compared to T0 and T9. T6 and T8 reduced hysteresis by 7% and 9%, k_F_ value by 8% and 11%, n value by 15% and 20%, k_L_ by 13% and 13%, and S_m_ by 29% and 37% compared to control at 25 °C and 35 °C (Table 2). Because the modifications in isotherm attributes resulted in a comparable drop in hysteresis and adsorption properties, T6 and T8 might be efficient in decreasing the adsorption of supplied P while simultaneously boosting the desorption of previously adsorbed P. Soil Si content was significantly improved by utilising rice straw and rice straw with phosphorus-solubilizing microbes. At 25 °C, T6 and T8 soil exhibited 64% and 73% greater Si contents than T0. A similar trend was seen at 35 °C (Table 2). Because treatments T6 and T8 transformed a bigger quantity of P, P solubilisation may increase, resulting in greater uptake by plants. Administration of rice straw alone at various dosages had a substantial influence on P transformation. Because P transformation with rice straw with phosphorus-solubilizing microbes was more successful than rice straw alone, based on Si release and the hysteresis effect, T6 and T8 were chosen for the next experiment to measure plant growth.

**Table 2:** Impact of rice straw and phosphorus-solubilizing microbes application on the parameters of Freundlich and Langmuir adsorption isotherms, phosphorus hysteresis (%), and silicon availability (Si; mg kg^−1^) in a semi-arid Inceptisol.

Temperature	25 °C						35 °C					
Treatments	Hysteresis	k_F_	n	k_L_	S_m_	Si	Hysteresis	k_F_	n	k_L_	S_m_	Si
T0	91.32 ± 8.67a	72.04 ± 6.71a	0.396 ± 0.03a	0.144 ± 0.03a	384.62 ± 23.5a	48.30 ± 5.23d	87.32 ± 8.24a	73.15 ± 6.49a	0.335 ± 0.03a	0.178 ± 0.04a	320.18 ± 21.68a	48.30 ± 5.11d
T1	90.46 ± 8.53a	71.18 ± 6.88ab	0.390 ± 0.04a	0.140 ± 0.02ab	370.71 ± 26.7b	52.10 ± 5.18cd	86.59 ± 8.36a	72.28 ± 6.43a	0.330 ± 0.03a	0.170 ± 0.06b	308.05 ± 23.47ab	54.77 ± 5.23d
T2	89.11 ± 8.42ab	69.78 ± 6.23bc	0.379 ± 0.03b	0.138 ± 0.02b	357.14 ± 24.2c	57.40 ± 5.29c	85.43 ± 8.54ab	70.86 ± 6.33ab	0.320 ± 0.02b	0.171 ± 0.05ab	296.22 ± 20.64b	62.60 ± 5.05c
T3	89.90 ± 8.61ab	70.50 ± 6.81ab	0.380 ± 0.06b	0.140 ± 0.03a	355.83 ± 25.6c	55.23 ± 5.27cd	86.10 ± 8.66a	71.59 ± 6.83ab	0.320 ± 0.02b	0.170 ± 0.03b	295.07 ± 26.46b	57.13 ± 5.34d
T4	87.42 ± 8.11bc	67.74 ± 6.44b	0.359 ± 0.04d	0.132 ± 0.02c	312.50 ± 24.7e	66.80 ± 5.73b	83.98 ± 8.13bc	68.79 ± 6.48b	0.304 ± 0.02c	0.163 ± 0.03c	257.28 ± 25.61d	69.70 ± 5.37b
T5	89.20 ± 8.23ab	69.90 ± 6.33bc	0.370 ± 0.06c	0.140 ± 0.02a	343.20 ± 24.6cd	59.4 ± 5.643	85.50 ± 8.61ab	70.99 ± 6.19ab	0.320 ± 0.03b	0.170 ± 0.03b	282.70 ± 23.52bc	62.43 ± 5.68c
T6	85.32 ± 8.34cd	65.96 ± 6.28c	0.336 ± 0.03e	0.126 ± 0.02d	274.62 ± 24.3f	79.40 ± 6.13a	82.19 ± 8.37bc	66.98 ± 6.28bc	0.284 ± 0.04d	0.156 ± 0.03cd	220.18 ± 23.49e	85.60 ± 6.23a
T7	88.59 ± 8.51ab	69.28 ± 6.15bc	0.370 ± 0.06c	0.140 ± 0.03a	332.96 ± 24.8d	60.83 ± 5.86bc	84.99 ± 8.76ab	70.36 ± 6.40ab	0.310 ± 0.03c	0.170 ± 0.03b	275.13 ± 23.44c	63.47 ± 5.64bc
T8	83.50 ± 8.09d	64.10 ± 6.24c	0.316 ± 0.04f	0.125 ± 0.02d	243.90 ± 24.6 g	83.60 ± 6.89a	80.64 ± 8.49c	65.09 ± 6.37c	0.266 ± 0.05e	0.154 ± 0.05d	197.46 ± 23.55f	88.70 ± 6.44a
T9	90.95 ± 8.76a	71.72 ± a6.79b	0.392 ± 0.07a	0.140 ± 0.03a	370.37 ± 27.6b	50.60 ± 5.06cd	87.01 ± 8.95a	72.83 ± 6.62a	0.332 ± 0.04a	0.173 ± 0.04ab	307.76 ± 26.83ab	53.40 ± 5.02d

Means with a similar lower-case letter within a column are not significantly different according to Tukey’s HSD test (*p* < 0.05).

### P transformation in the soil during different growth stages of wheat

**Figure 2:**
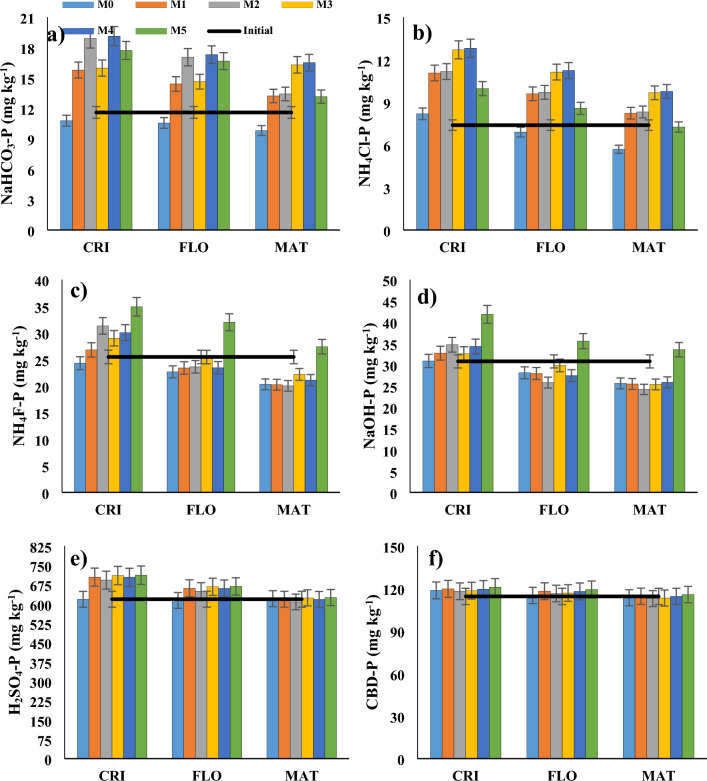
Changes in (**a**) plant available P (NaHCO_3_-P), (**b**) ammonium chloride extractable P (NH_4_Cl-P), (**c**) ammonium fluoride extractable P (NH_4_F-P), (**d**) sodium hydroxide extractable P (NaOH-P), (**e**) sulfuric acid extractable P (H_2_SO_4_-P) and (**f**) citrate-bicarbonate extractable P (CBD-P) in the soil during crown root initiation (CRI), flowering (FLO), and maturity (MAT) stages of wheat in a semi-arid subtropical Inceptisol. Error bars indicate LSD (*p* = 0.05).

At the crown root initiation growth stage, M2 and M4 exhibited 75% and 77% greater NaHCO_3_-P, and 36% and 56% greater NH_4_Cl-P, respectively (Fig. 2a,b). Similar patterns were seen during the flowering and maturation periods of wheat. M2 and M4 solubilised only a small quantity of NH_4_F-P during the crown root initiation phase. Under M2 and M4, there was net NH_4_F-P fixation, however, during this flowering period, they solubilized 7.3% and 7.9% NH_4_F-P, respectively (Fig. 2c). At the maturation stage, M2 and M4 dissolved 21% and 17% of NH_4_F-P, respectively. M2 and M4 dissolved 16% and 10% NaOH-P at the flowering stage, and 21 and 16% NaOH-P during the maturation stage, respectively (Fig. 2d). Following earlier fixation in the crown root initiation stage, progressive solubilisation of H_2_SO_4_-P was detected during the flowering and maturation stages (Fig. 2e). The citrate-bicarbonate extractable P, on the other hand, did not vary appreciably throughout crop growth, even though some P was fixed as citrate-bicarbonate extractable P at the crown root initiation and flowering stages (Fig. 2f). After initial P entrapment at the crown root initiation stage, consistent hydrolysis of organic P was detected during the flowering and maturity stages (Fig. 4a).

### P transformation in the soil during different growth stages of rice

**Figure 3:**
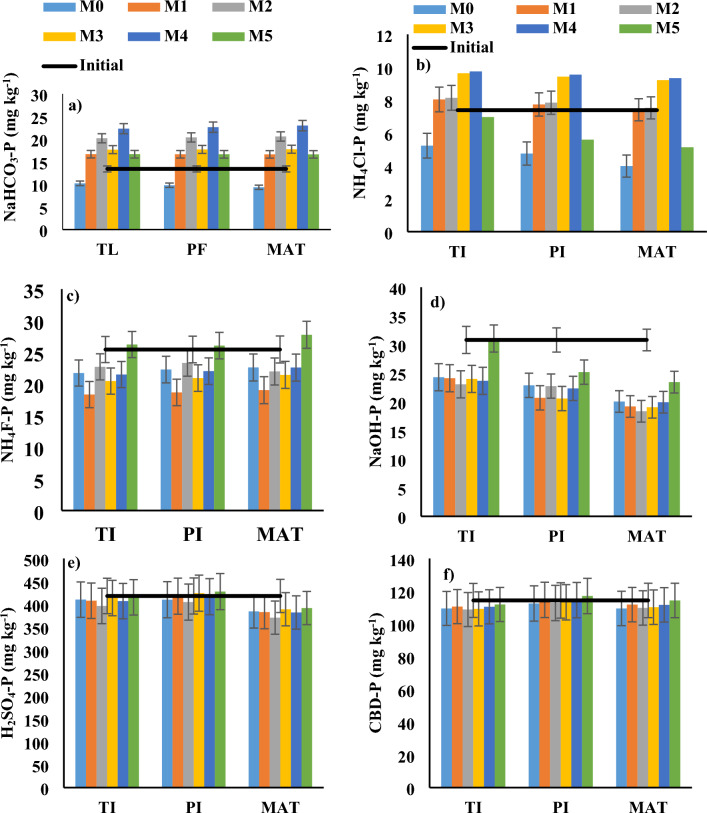
Changes in (**a**) plant available P (NaHCO_3_-P), (**b**) ammonium chloride extractable P (NH_4_Cl-P), (**c**) ammonium fluoride extractable P (NH_4_F-P), (**d**) sodium hydroxide extractable P (NaOH-P), (**e**) sulfuric acid extractable P (H_2_SO_4_-P) and (**f**) citrate-bicarbonate extractable P (CBD-P) in the soil during tiller initiation (TI), flowering (FLO), and maturity (MAT) stages of rice in a semi-arid subtropical Inceptisol. Error bars indicate LSD (*p* = 0.05).

At the TI stage, M2 and M4 had ~ 40 and 75% higher plant available P than M0, respectively. Similarly, at the flowering stage of rice, M2 and M4 had ~ 45 and 85% higher plant available P than M0, respectively (Fig. 3a). At maturity stage of rice, M2 and M4 had ~ 51 and 95% higher plant available P than M0. The NH_4_Cl-P under M2 and M4 was ~ 56 and 86% higher than M0 and 17 and 40% higher than M5, respectively, at tiller initiation stage of rice. The NH_4_Cl-P under M2 and M4 was ~ 65 and 80% higher than M0 and 40 and 71% higher than M5, respectively, at panicle initiation stage of rice. The NH_4_Cl-P under M2 and M4 was ~ 63 and 83% higher than M0 and 47 and 82% higher than M5, respectively, at the maturity stage of rice (Fig. 3b). The NH_4_F-P under M2 and M4 was similar to M0 during tiller initiation, panicle formation, and maturity stages of rice. The NH_4_F-P under M2 and M4 was 13 and 18% lower than M5, respectively, at tiller initiation stage of rice, 11 and 16% lower than M5, respectively, at panicle initiation stage of rice, and ~ 21 and 19% lower than M5, respectively, at maturity stage of rice (Fig. 3c). The NaOH-P under M2 and M4 was similar to M0 during tiller initiation, panicle formation, and maturity stages of rice. The NaOH-P under M2 and M4 was 26 and 24% lower than M5, respectively, at tiller initiation stage of rice, 10 and 11% lower than M5, respectively, at panicle initiation stage of rice, and ~ 22 and 15% lower than M5, respectively, at maturity stage of rice (Fig. 3d). The H_2_SO_4_-P under M2 and M4 was similar to M0 and M5 during tiller initiation, panicle formation, and maturity stages of rice (Fig. 3e). The CBD-P did not change much by application of RS + PSM during the three stages of rice growth (Fig. 3f). The organic P under M2 and M4 was ~ 13 and 23% higher than M0, respectively at tiller initiation stage of rice. The organic P under M2 and M4 was ~ 21 and 27% higher than M0, respectively, at panicle initiation stage of rice. The organic P under M2 and M4 was ~ 22 and 29% higher than M0 respectively, at maturity rice. The organic P under M2 and M5 was similar during all three growth stages of rice. However, M4 had ~ 6, 7, and 6% greater organic P than M5 during those corresponding growth stages of rice (Fig. 6a).

### Activities of soil enzymes and organic acids in wheat rhizosphere

**Figure 4:**
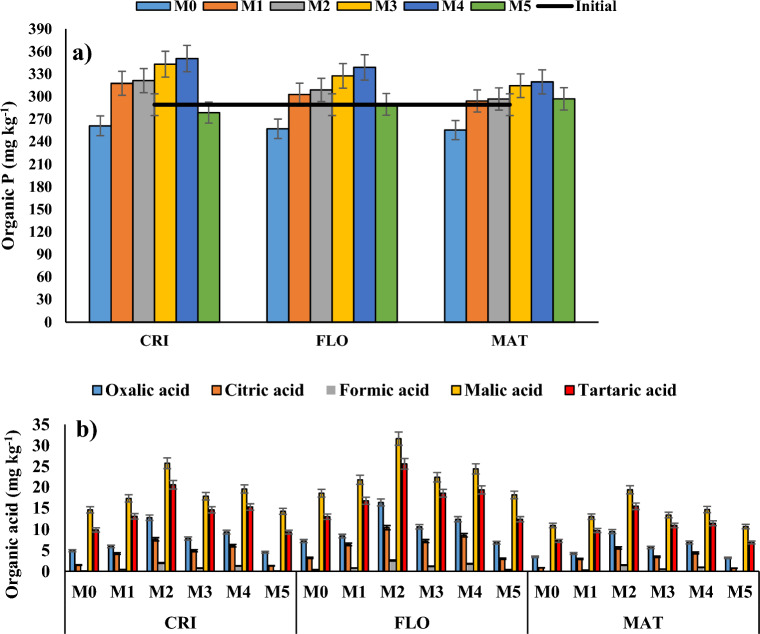
Changes in (**a**) soil organic P and (**b**) organic acid concentrations in the soil during crown root initiation (CRI), flowering (FLO), and maturity (MAT) stages of wheat in a semi-arid subtropical Inceptisol. Error bars indicate LSD (*p* = 0.05).

The five organic acids measured were oxalic acid, citric acid, formic acid, malic acid, and tartaric acid. The maximum level of every organic acid was found during the flowering period of wheat, followed by the crown root initiation and mature stages (Fig. 3b). Under M2, the quantity of organic acids was significantly higher than other management strategies in all stages of wheat. However, in all stages of wheat, M2 and M4 exhibited considerably higher organic acid contents than M5 and M0 (Fig. 4b). At the crown root initiation stage, oxalic acid, citric acid, formic acid, malic acid, and tartaric acid levels were 2.8, 5.8, 21.1, 1.8, and 2.2 times higher than M5, respectively. Similar patterns were seen during the flowering and maturation periods of wheat.

**Figure 5:**
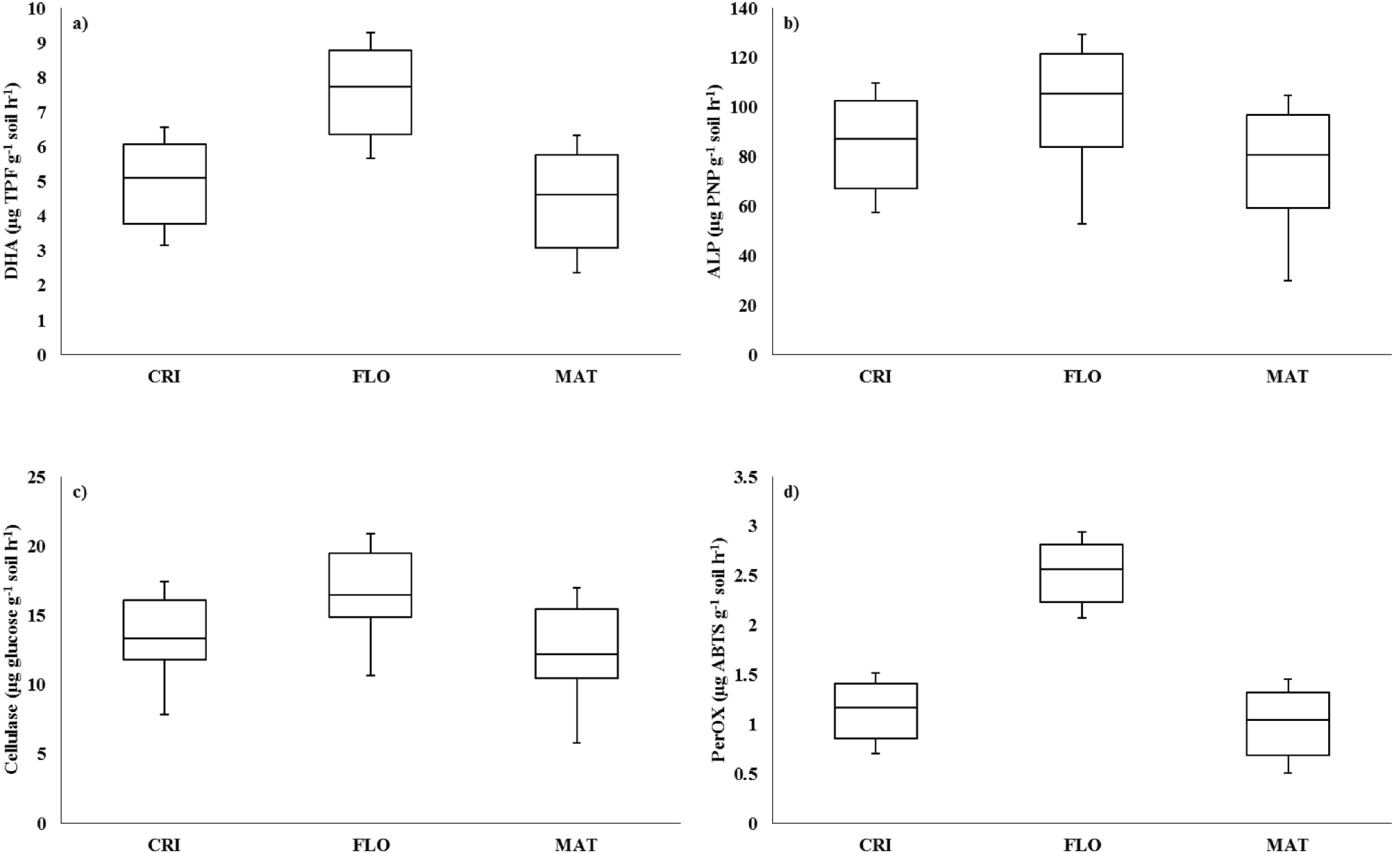
Changes in (**a**) dehydrogenase (DHA), (**b**) alkaline phosphatase (ALP), (**c**) cellulase, and (**d**) peroxidase (PerOX) activity in the soil during crown root initiation (CRI), flowering (FLO), and maturity (MAT) stages of wheat in a semi-arid subtropical Inceptisol.

During the flowering stage, the maximum level of activity for each enzyme was measured. The addition of rice straw with phosphorus-solubilizing microbes increased activity of soil enzymes over M0 and M5. During the crown root initiation stage, the activities of dehydrogenase, alkaline phosphatase, cellulase, and peroxidase enhanced by 1.59, 1.50, 1.25, and 1.62 times, correspondingly, under M2 over M5 (Fig. 5a–d). However, the activities of dehydrogenase, alkaline phosphatase, cellulase, and peroxidase improved by 1.87, 1.75, 1.48, and 1.91 times, respectively, when M4 was used over M5 during the crown root initiation stage of wheat. Similar patterns were identified during the flowering and maturation periods of wheat (Fig. 5a–d).

### Activities of soil enzymes and organic acids in rice rhizosphere

**Figure 6:**
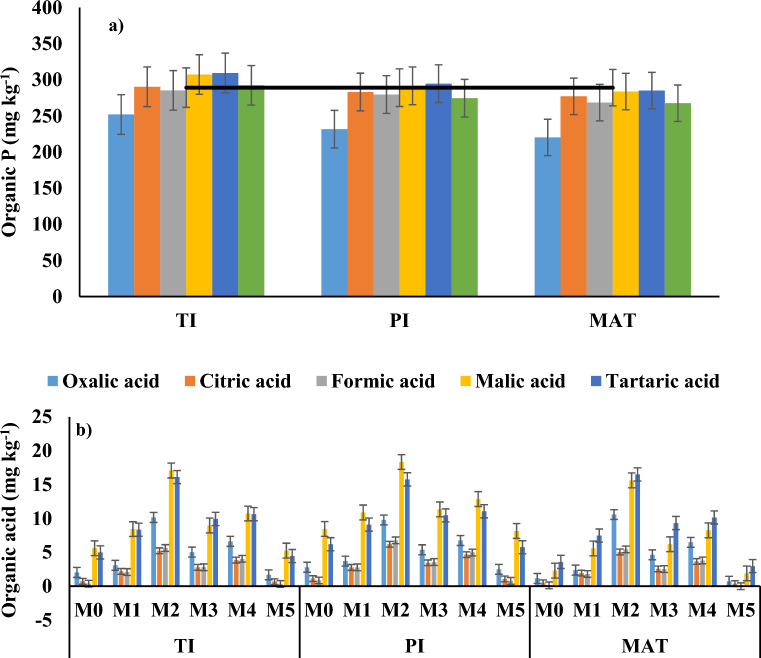
Changes in (**a**) soil organic P and (**b**) organic acid concentrations in the soil during tiller initiation (TI), flowering (FLO), and maturity (MAT) stages of rice in a semi-arid subtropical Inceptisol. Error bars indicate LSD (*p* = 0.05).

**Figure 7:**
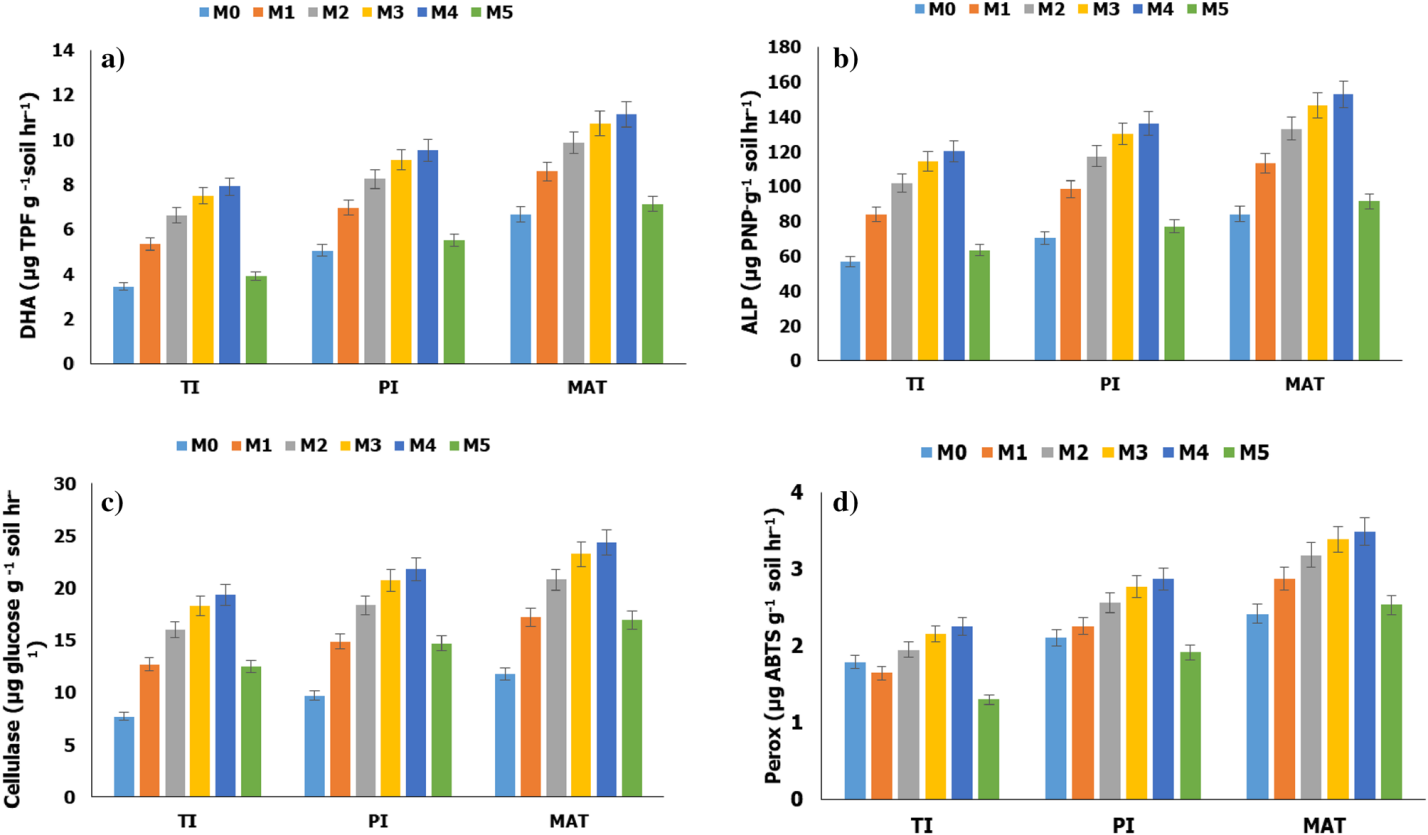
Changes in (**a**) dehydrogenase (DHA), (**b**) alkaline phosphatase (ALP), (**c**) cellulase, and (**d**) peroxidase (PerOX) activity in the soil during tiller initiation (TI), flowering (FLO), and maturity (MAT) stages of rice in a semi-arid subtropical Inceptisol.

At the tiller initiation stage of rice, M2 and M4 had ~ 6.01 and 3.92 folds greater oxalic acid than M5, 5.74 and 4.32 folds greater citric acid than M5, 8.44 and 6.20 folds greater formic acid than M5, 2.25 and 1.58 folds greater malic acid than M5, and 2.74 and 1.92 folds greater tartaric acid than M5, respectively. The identical trends persisted during panicle initiation and maturity stages of rice (Fig. 6b). Dehydrogenase activity under M2 and M4 was ~ 37 and 101%, 26 and 72%, and 20 and 56% greater than M5, respectively during tiller initiation, panicle initiation and maturity stages of rice. Alkaline phosphatase activity under M2 and M4 was ~ 32 and 89%, 28 and 77%, and 24 and 67% greater than M5, respectively during tiller initiation, panicle initiation and maturity stages of rice. Similar trends were also observed for cellulase, and peroxidase activities (Fig. 7).

### Si availability and pH in the soil during different crop growth stages

The use of rice straw with phosphorus-solubilizing microbes over control and M5 substantially enhanced Si levels in the soil. It also grew steadily in crown root initiation, blooming, and maturity stages (Table 3). M2 and M4 contained 17 and 21%, 43 and 47%, and 58 and 63% higher Si contents at crown root initiation, flowering, and maturation stages of wheat, respectively, than M0. During different phases of wheat growth, the Si content in the soil under M0 and M5 did not vary appreciably. M2 and M4 improved Si concentration by ~ 39 and 43% during tiller initiation stage, 34 and 39% during panicle initiation stage, 15 and 19% during maturity stage of rice, over M5, respectively (Table 3). Rice straw with phosphorus-solubilizing microbes drastically decreased soil pH compared to control and M5 throughout crop growth. M2 and M4 retained a 10% lower soil pH than M0 and a 7% lower soil pH than M5 in wheat rhizosphere. However, M2 and M4 had ~ 5–11% lower pH than M0 and M5 during the growth stages of rice. The concentrations of organic acids were positively correlated with the reduction in soil pH.

**Table 3:** Impact of rice straw and phosphorus-solubilizing microbes application on silicon availability (Si; (mg kg^−1^)) and pH in the soil during different growth stages of wheat and rice in a semi-arid Inceptisol.

Crop	Si (mg kg^−1^)			pH		
	Wheat					
Management strategy	*CRI	FLO	MAT	CRI	FLO	MAT
M0	48.27 ± 5.07c	45.28 ± 5.44c	43.41 ± 5.06c	8.09 ± 0.64a	8.08 ± 0.73a	8.09 ± 0.71a
M1	51.52 ± 5.16bc	58.96 ± 5.31b	63.02 ± 5.63b	7.38 ± 0.52c	7.35 ± 0.62c	7.40 ± 0.64c
M2	56.48 ± 5.23ab	64.56 ± 5.67ab	68.51 ± 5.34ab	7.34 ± 0.56cd	7.33 ± 0.53cd	7.36 ± 0.63cd
M3	54.48 ± 5.34b	62.30 ± 5.88ab	66.29 ± 5.87ab	7.29 ± 0.59d	7.26 ± 0.58d	7.29 ± 0.67d
M4	58.34 ± 5.19a	66.67 ± 5.94a	70.57 ± 6.10a	7.27 ± 0.61d	7.25 ± 0.61d	7.28 ± 0.66d
M5	49.35 ± 5.04c	46.50 ± 5.13c	44.61 ± 5.11c	7.90 ± 0.63b	7.79 ± 0.54b	7.84 ± 0.68b

Crop	Si (mg kg^−1^)			pH		
	Rice					
	TI	FLO	MAT	CRI	FLO	MAT
M0	43.70 ± 5.01c	41.17 ± 5.11c	36.60 ± 4.64c	8.04 ± 0.68a	7.86 ± 0.67a	8.26 ± 0.76a
M1	57.10 ± 5.63b	49.24 ± 5.07b	39.19 ± 4.28b	7.71 ± 0.53bc	7.16 ± 0.83b	7.55 ± 0.63b
M2	62.28 ± 5.34ab	53.91 ± 5.34ab	43.13 ± 4.39a	7.67 ± 0.51cd	7.12 ± 0.54b	7.51 ± 0.68b
M3	60.19 ± 5.28ab	52.02 ± 5.26ab	41.53 ± 5.02ab	7.60 ± 0.59d	7.05 ± 0.61b	7.44 ± 0.61b
M4	64.23 ± 5.83a	55.67 ± 5.29a	44.61 ± 4.34a	7.58 ± 0.68d	7.03 ± 0.49b	7.43 ± 0.63b
M5	44.83 ± 5.06c	40.19 ± 5.03c	37.46 ± 4.52c	7.86 ± 0.39ab	7.91 ± 0.68a	7.56 ± 0.62b

Means with a similar lower-case letter within a column are not significantly different according to Tukey’s HSD test (*p* < 0.05).

*crown root initiation (CRI), flowering (FLO), and maturity (MAT) stages of wheat and tiller initiation (TI), flowering (FLO), and maturity (MAT) stages of rice.

### Root traits, yield and P uptake of wheat

Treatment M2 gave the greatest increase in grain yield. Grain production under M2 and M4 was 40% and 27% greater than M0, and 18% and 8% greater than M5, respectively. Wheat straw output under M2 and M4 was 1.39 and 1.26 times greater than M0, and 1.18 and 1.07 times greater than M5, respectively (Table 4). P acquisition by grain under M2 and M4 increased by 91% and 32% over M0, and 72 and 19% over M5, respectively. Total P absorption by wheat improved by 36% and 19% under M2 and M4, respectively, over control (Table 4). Wheat root characteristics exhibited a close linkage with P acquisition. Wheat root length with M2 and M4 was 1.78 and 1.22 times larger than M0 at the crown root initiation stage, 1.88 and 1.37 times bigger than M0 at the flowering phase, and 1.74 and 1.32 times higher than M0 at the maturity stage, respectively (Table 4). At the crown root initiation stage, the root volume of wheat under M2 and M4 was 2.68 and 2.14 times higher than M0, respectively. At the flowering phase, root volume was 2.50 and 2.17 times higher than M0, respectively. At the mature stage, root volume was 2.38 and 2.13 times greater than M0, respectively. In all three development phases, M2 and M4 developed larger root lengths and volumes of wheat than M5.

**Table 4:** Impact of rice straw and phosphorus-solubilizing microbes application on the grain and straw yield (mg pot^−1^), P uptake (mg pot^−1^), and root length (cm plant^−1^) and volume (cm^3^ plant^−1^) of wheat in a semi-arid Inceptisol.

Management strategy	Yield		P uptake		Total root length			Root volume		
	Wheat									
	Grain	Straw	Grain	Straw	*CRI	FLO	MAT	CRI	FLO	MAT
M0	6.19 ± 0.54c	9.64 ± 1.03c	14.79 ± 1.64e	5.24 ± 0.44e	159 ± 11f	175 ± 18e	224 ± 19f	63 ± 7e	75 ± 10e	87 ± 9e
M1	8.21 ± 0.63a	12.77 ± 1.24ab	22.69 ± 1.79b	7.87 ± 0.63b	265 ± 13b	311 ± 22b	371 ± 23b	138 ± 11c	157 ± 12c	179 ± 16c
M2	8.67 ± 0.67a	13.48 ± 1.06a	28.24 ± 1.67a	8.47 ± 0.68a	282 ± 15a	328 ± 24a	389 ± 24a	167 ± 16a	187 ± 16a	208 ± 18a
M3	7.69 ± 0.64b	11.97 ± 1.06b	17.39 ± 1.62d	7.19 ± 0.67c	226 ± 12c	272 ± 21c	330 ± 22c	148 ± 12b	169 ± 15b	195 ± 17b
M4	7.87 ± 0.66b	12.25 ± 1.26ab	19.51 ± 1.39c	7.43 ± 0.69c	193 ± 20d	239 ± 20d	296 ± 26e	134 ± 10c	163 ± 16bc	185 ± 16bc
M5	7.31 ± 0.67b	11.38 ± 1.06b	16.45 ± 1.35d	6.70 ± 0.59d	171 ± 16e	262 ± 25c	320 ± 28d	116 ± 15d	130 ± 11d	155 ± 13d

Management strategy	Yield		P uptake		Total root length			Root volume		
	Rice									
	Grain	Straw	Grain	Straw	*TI	FLO	MAT	TI	FLO	MAT
M0	4.6 ± 0.51d	6.2 ± 0.64c	8.4 ± 1.61d	5.9 ± 0.68d	108 ± 13d	11,712 ± d	146 ± 13d	40 ± 8d	45 ± 8e	51 ± 7d
M1	8.7 ± 0.64c	11.4 ± 0.63b	20.9 ± 1.83c	9.6 ± 0.89c	170 ± 16a	197 ± 18a	232 ± 18a	72 ± 7b	79 ± 9c	88 ± 9b
M2	10.5 ± 0.69a	13.7 ± 0.61a	26.3 ± 4.96a	12.1 ± 1.06a	180 ± 20a	207 ± 19a	242 ± 20a	83 ± 9a	92 ± 10a	100 ± 10a
M3	9.1 ± 0.57b	11.9 ± 0.62b	22.1 ± 2.04b	10.2 ± 1.04bc	147 ± 15b	174 ± 16b	208 ± 19b	76 ± 7ab	84 ± 9b	95 ± 9ab
M4	10.2 ± 0.63a	13.3 ± 058a	25.5 ± 2.16a	11.8 ± ab	128 ± 12c	155 ± 14c	188 ± 16c	70 ± 7b	82 ± 8bc	91 ± 8ab
M5	9.6 ± 0.53b	12.5 ± 0.54ab	23.6 ± 2.11b	10.9 ± 0.89b	115 ± 14d	168 ± 17bc	202 ± 17b	62 ± 8c	68 ± 9d	78 ± 8c

Means with a similar lower-case letter within a column are not significantly different according to Tukey’s HSD test (*p* < 0.05).

*crown root initiation (CRI), flowering (FLO), and maturity (MAT) stages of wheat and tiller initiation (TI), flowering (FLO), and maturity (MAT) stages of rice.

### Root traits, yield and P uptake of rice

M2 and M4 improved rice root length by ~ 67 and 19% during tiller initiation stage, 76 and 32% during panicle initiation stage, 66 and 29% during maturity stage of rice over M0, respectively. M2 and M4 improved rice root volume by ~ 2.06 and 1.73 folds during tiller initiation stage, 2.02 and 1.80 folds during panicle initiation stage, 1.98 and 1.80 folds during maturity stage of rice, over M0 respectively (Table 4). Rice grain production under M2 and M4 was 25 and 20% greater than M0, respectively, and 9 and 6% greater than M5. Rice straw output under M2 and M4 was 1.22 and 1.16 times greater than M0, respectively, and 1.9 and 1.06 times greater than M5, respectively. Phosphorus acquisition by rice grain under M2 and M4 increased by 3.12 and 3.03 folds greater over M0, and 12 and 8% over M5, correspondingly. Phosphorus uptake by rice straw under M2 and M4 was ~ 2.06 and 1.99 folds greater than M0, and 11 and 8% greater than M5, respectively. Total P uptake by rice under M2 and M4 improved by 2.68 and 2.60 times over control, and 12 and 8% over M5, respectively (Table 4).

### Interrelationship of available phosphorus with other soil parameters

Soil available P (NaHCO_3_-P) was positively correlated (R^2^ > 0.768, *p* < 0.05) with saloid-bound P during all stages of wheat. Among other fractions of inorganic soil P, NH_4_F-P (R^2^ > 0.678, *p* < 0.05) and NaOH-P (R^2^ > 0.713, *p* < 0.05) were significantly correlated with NaHCO_3_-P at the flowering and maturity stages. However, H_2_SO_4_-P and CBD-P were not correlated with available P in any stage of plant growth. The concentration of Si in soil was positively correlated (R^2^ > 0.793, *p* < 0.05) with available P during all growth stages. However, the concentrations of organic acids were not correlated with available P in any stages of plant growth. Concentrations of organic acids were significantly correlated with root length (R^2^ > 0.729, *p* < 0.05) and volume (R^2^ > 0.716, *p* < 0.05) of in all stages of growth. The dehydrogenase activity in soil was positively correlated (R^2^ > 0.847, *p* < 0.05) with available P during all growth stages. The ALP activity in soil positively contributed (R^2^ > 0.821, *p* < 0.05) to available P during all growth stages. Cellulase and peroxidase activities in soil were positively correlated (R^2^ > 0.868 and R^2^ > 0.846, respectively; *p* < 0.05) with available P during all plant growth stages.

## Discussion

Due to the greater stability of H_2_SO_4_-P at the soil’s initial pH value (8.2), a greater amount of NaOH-P and NH4F-P could be mobilised by rice straw with phosphorus-solubilizing microbes at 25 °C. Furthermore, the introduction of rice straw with phosphorus-solubilizing microbes led to a significant substitution of PO_4_^3−^ by SiO_4_^4−^ in Fe-phosphate and Al-phosphate minerals, causing a release of PO_4_^3−^ into the soil solution. The NaOH-P and NH_4_F-P could be mobilised by ligand replacement of PO_4_^3−^ by SiO_4_^4−^, dissolving of Fe/Al-phosphate minerals by organic acids, and other processes. The H_2_SO_4_-P is liberated during the breakdown of Ca-phosphate minerals by organic acids. The surge in NH_4_Cl-P could be attributed to the sorption of solubilised and hydrolysed P on soil particles. Previous research suggested that phosphorus-solubilizing microbes and Si might also help plants flourish in low-P environments^17,18^. Phosphorus-solubilizing microbes worked successfully in the cycles of dissolution–precipitation, mineralization-immobilisation, and sorption–desorption for transforming insoluble phosphates bioavailable^19^. Organic acids, through ligand-induced dissolution, might remove P from mineral surfaces. Organic acids either competed with or displaced adsorbed phosphates for fixation sites on soil clay particles such as amorphous aluminium oxides, goethite, kaolinite, and montmorillonite^20^. Organic acids helped in the liberation of NH_4_F-P, NaOH-P, and H_2_SO_4_-P by increasing the chelation with P-bound to Al^3+^, Ca^2+^, and Fe^3+^ or by forming soluble metal ion complexes and inhibiting P precipitation. The inclusion of rice straw with phosphorus-solubilizing microbes enhanced the quantities of SiO44- ions in soil solution. SiO_4_^4−^ ions and organic acids contended for adsorbent surface with phosphate ions in the solution, resulting in a significant decrease in k_F_ (for Freundlich isotherm), k_L_, and S_m_ (for Langmuir isotherm) for P sorption. At a higher temperature, the quantity of SiO_4_^4−^ ions and organic acids increased, and rice straw with phosphorus-solubilizing microbes substantially reduced the adsorption behaviour (such as k_F_ and S_m_) to enhance the amount of NaHCO_3_-P. (Table 2).

Organic anions, according to Afif et al.^21^, could compete with phosphorous for a similar adsorbent surface, reducing P sorption. This emphasised the necessity for SiO_4_^4−^ and organic acids to impede P adsorption. The use of rice straw raised the concentration of SiO_4_^4−^ ions as well (Table 2). The adsorbent surface could also exchange PO_4_^3−^ ions for SiO_4_^4−^ ions. This was understandable given that P adsorption could fall while Si adsorption might be nearly at its peak in the pH range of 6–11. These outcomes indicated that the soil pH needed to be relatively high (> 7.0) for silicate treatment to properly restrict phosphate binding. Indeed, the utilisation of rice straw with phosphorus-solubilizing microbes liberated organic acids and Si, as well as aided in the mobilisation of inorganic P. As indicated by increasing NH_4_Cl-P at 35 °C, phosphate adsorption decreased with increasing silicate concentration and temperature in the solution (Fig. 1a,b). At all temperatures, there was a significant relationship between the amount of phosphate mobilised from NaOH-P and NH_4_F-P and silica content (R^2^ > − 0.823; *p* < 0.05), but organic acid concentration was strongly related to H_2_SO_4_-P (R^2^ > − 0.769; *p* < 0.05). This demonstrated that NaOH-P and NH_4_F-P mobilisation was influenced by the liberation of PO_4_^3−^ ions by SiO_4_^4−^, whereas H_2_SO_4_-P solubilisation was impacted by the solvation of Ca-phosphate minerals through organic acids. In the incubation experiment, T6 and T8 could boost inorganic P mobilisation to enhance availability by solubilizing 3.4–3.7% of fixed P. (Fig. 1a,b). As a result, they might be employed for agricultural production. T6 and T8 have been identified for the second experiment following the outcomes of the incubation study to confirm their performance during crop production.

Greater activity of soil enzymes such as dehydrogenase, ALP, and cellulase under M1, M2, M3, and M4 compared to M0 and M5 could be attributed to higher substrate accessibility due to rice straw (Fig. 4a–d). Cellulose generated through rice straw breakdown served as a substrate for cellulase enzymes in the soil^22^. Greater cellulose concentrations in M2 and M4 in the presence of rice straw may have boosted their activity when compared to M0 and M5^23^. Peroxidase transforms resistant aromatic molecules like lignin into more labile substrates^24^. Undoubtedly, enhanced peroxidase enzyme activity in M2 and M4 over M0 and M5 were related to increased substrate availability by rice straw with phosphorus-solubilizing microbes. According to Ghosh et al.^24^, crop residues influence phosphatase activity in the soil. There was a strong connection between residue application and phosphatase activity in the soil of the current investigation. Increased enzyme activity resulted in quicker breakdown of rice straw, resulting in a considerable increase in organic acid concentration in the soils of the rhizosphere. Increased organic acid concentrations resulted in a decrease in pH in the soil under M2, M3, and M4 compared to M0 and M5. The breakdown of rice straw resulted in greater Si concentration in the soil under M2, M3, and M4 over M0 and M5. Si increased soil P availability and plant P uptake by mobilising or desorbing organic P from soil particles or mineral binding sites (e.g., goethite) via competitive exchange and P-Si sorptive interaction^25^. Organic acid-mediated decomposition assisted in the liberation of Al/Fe/Ca bound P. Mobilisation of P from Al/Fe/Ca-phosphate compound resulted in a significant increase in saloid-bound P in M2, M3, and M4 than M0 and M5.

**Figure 8:**
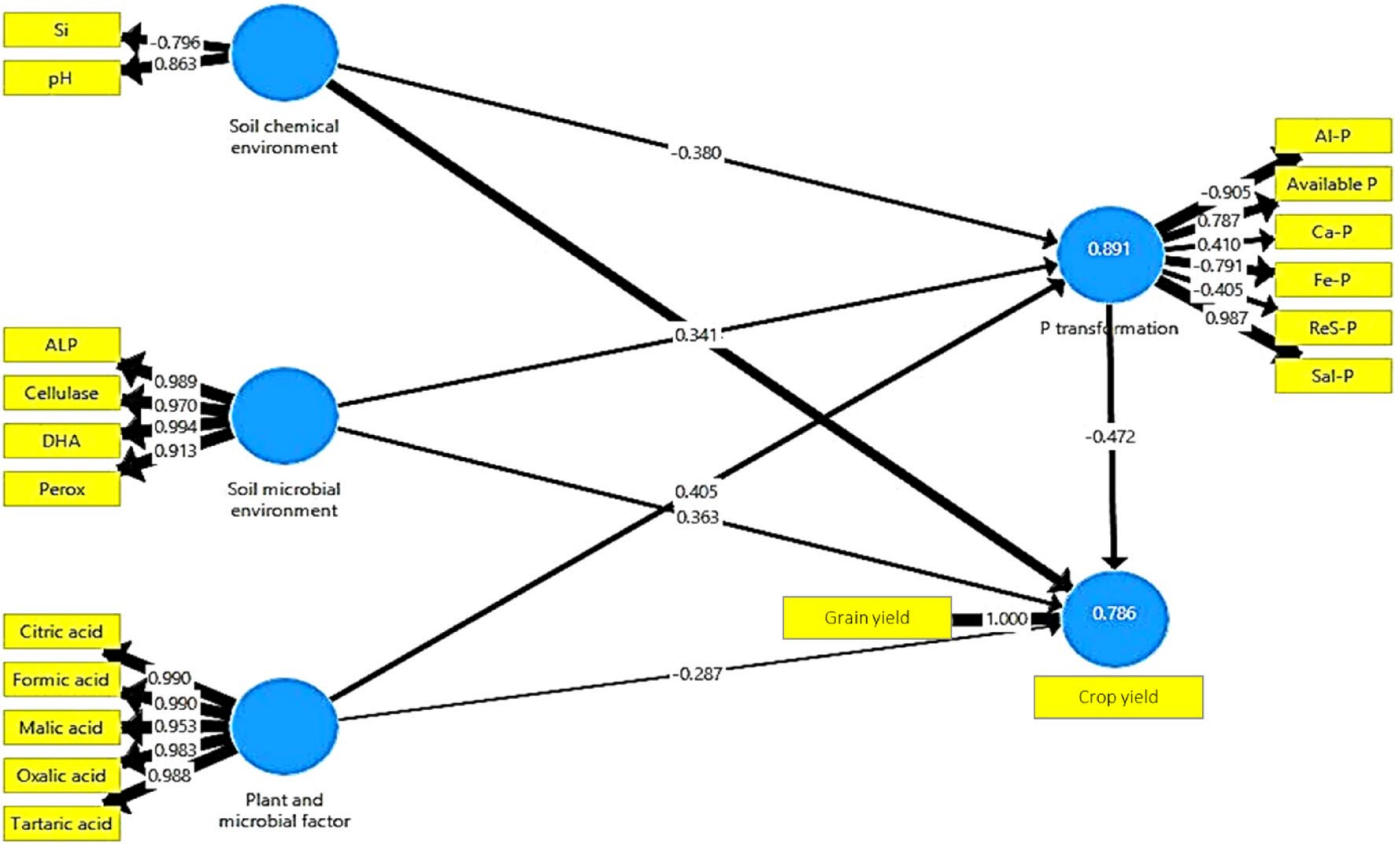
Path analysis to depict the impacts of soil chemical environment, soil microbial environment, and plant and microbial factors on phosphorus (P) mobilisation in the soils of the wheat rhizosphere. Models satisfactorily fitted to data based on χ2 and RMSEA analyses [χ2 = 341.37, GFI = 0.92, RMSEA < 0.001]. Solid arrows represent the significant effects. The width of the arrows indicates the strength of the casual relationship. Si: Silicon, pH: soil pH, ALP: Alkaline phosphatase, DHA: Dehydrogenase; PerOX: peroxidase; Org P: Organic phosphorus mineralized; NH_4_Cl-P: Ammonium chloride extractable P, NH_4_F-P: Ammonium fluoride extractable P, NaOH-P: Sodium hydroxide extractable P; CBD-P: Citrate-bicarbonate extractable P, H_2_SO_4_-P: Sulfuric acid extractable P.

Higher P bioavailability resulted in its increased acquisition by wheat straw and grain. The structural equation modelling of soil parameters clearly highlighted changes in the mechanistic routes for enhancing P availability. The structural equation model (SEM) revealed that soil pH and Si concentration dominated the soil chemical environment (Fig. 8). The chemical environment of the soil had a substantial impact on the microbiological ecology, and vice versa. Furthermore, the chemical and microbiological environments of the soil had a substantial influence on the release of organic acids. The SEM demonstrated that the chemical environment of the soil, enzyme activity, and organic acids all had a significant influence on P transformation in the soil (Fig. 5). Higher soil enzyme activity resulted in quicker degradation of rice straw, increasing the amounts of Si and organic acids in the soil under M2, M3, and M4^26^. The higher the Si content in the soil, the more competitive desorption of phosphate by silicate took place to liberate previously absorbed P. The pH of the soil was regulated at 7–8. Si adsorption inhibits phosphate adsorption in the pH range of 6–11. Furthermore, the organic acids developed complexes with ionic species of Fe, Al, or Ca, releasing phosphate from iron, aluminium, and calcium phosphate compounds. This is substantiated by the decrease in NH4F-P, NaOH-P, and H_2_SO_4_-P in the soil under M2, M3, and M4 (Fig. 2c–f). These mechanisms may also have minimized P hysteresis, enabling the release of adsorbed P, as seen in the first experiment. This is substantiated by the fact that NH_4_F-P, NaOH-P, and H_2_SO_4_-P levels increased significantly at the crown root initiation stage and subsequently reduced during wheat flowering and maturity under M2, M3, and M4 (Fig. 2c–f). By looking at the higher coefficients, it is apparent that the solubilized P moved mostly to NH_4_Cl-P and NaHCO_3_-P. (Fig. 8). Importantly, the significant negative coefficient for organic P indicated that the influence of soil organic P on NH_4_Cl-P and NaHCO_3_-P was also considerable (Fig. 8). The transformation of P had an influence on wheat production by improving P absorption.

P availability has a substantial influence on wheat productivity. Even though M3 and M4 had higher levels of rice straw, M2 delivered the best yield and the maximum P absorption by wheat. This might be related to the initial immobilisation of nutrients under M3 and M4 due to the use of a high amount of rice straw, which impeded crop development substantially, lowering production and P absorption. PSM is readily available in market at a very low cost and RS can be obtained from the farmers’ field itself. This technology additionally saves 25% P fertilizer and enhances the crop yield. Hence, this technology could be economically feasible for the resource poor farmers.

P acquisition was strongly influenced by root properties, which included root length and root volume. Greater root volume and length under M2 enabled the root system to traverse more soil and offered a higher surface area for mineral nutrient intake^27^. For much less mobile PO_4_^3^ ion, a bigger root system provided a higher absorption surface^28^. The synchronised proliferation of roots at all growth phases might be the cause of wheat’s greater root efficiency under M2. It seemed that a root system’s growth facilitated improved P absorption, which supported higher biomass output. Despite M4 having a larger P concentration in the soil than M2, M2 had a stronger P uptake by wheat. P uptake was dependent on both the root system’s ability to absorb nutrients and the above-ground growth that resulted in the demand for nutrients in the shoot, suggesting that P uptake was driven by increased shoot demand under M2. Our study demonstrated the functional relationships between root features and P absorption by finding significant positive associations between root volume (R^2^ = 0.786 and 0.739, respectively; *p* < 0.05) and length (R^2^ = 0.713 and 0.724, respectively; *p* < 0.05) with P concentration in shoot and grain. However, the study has some limitations, as we did not test this technology on farm and did not actually evaluate the cost and benefit of it.

## Conclusions

In this study an incubation and pot experiment was conducted to assess the potential of RS application as Si source for solubilising inorganically fixed P. We also assessed P release kinetics, enzyme activity, and organic acid production and employed a path model to understand the mechanisms. Here, we showed in the first experiment that adding rice straw to subtropical soils at rates of 12 and 14 Mg ha^−1^ with phosphorus-solubilizing microbes is a practical way to solubilize the inorganic resistant soil P and minimize the hysteresis impact of P. Between 3.1 and 3.5% of the soil’s inorganic P may be solubilized by applying rice straw at rates of 12 and 14 Mg ha^−1^. It suggests that applying rice straw at a rate of 12 and 14 Mg ha^−1^ would be advantageous for supplying the P requirements of crops in semi-arid subtropical Inceptisols.

The inference from the first experiment was confirmed by the second experiment. It showed that the highest wheat yield was obtained when rice straw was applied at a rate of 12 Mg ha^−1^ along with phosphorus-solubilizing microbes and 75% mineral P fertiliser. Additionally, it made it easier for wheat to absorb P. Furthermore, by leaving more NaHCO_3_-P in the soil, it could improve the availability of P for subsequent crops. Provision of rice straw at a rate of 12 Mg ha^−1^ in conjunction with phosphorus-solubilizing microbes allowed soil pH to be maintained in a range close to neutral and, more crucially, increased the activities of soil enzymes. Because better yield and P acquisition were found with the application of rice straw at 12 Mg ha^−1^ with phosphorus-solubilizing microbes and 75% mineral P fertiliser, farmers can reduce P fertiliser costs by 25% using this strategy. This might lessen India’s total burden for P imports from other nations, when applied on a broader scale.

## Methods

### Soil and rice straw

The soil used in these experiments was sourced from the ICAR-Indian Agricultural Research Institute’s research farm in New Delhi, India (28° 37′–28° 39′ N, 77° 9′–77° 11′ E, 220 m above mean sea level). This soil is classified as *Typic Haplustept*, which is an Indo-Gangetic plains alluvial association^29^. The soil is a non-calcareous alkaline sandy loam with low cation exchange capacity. The study area has a semi-arid, subtropical climate characterized by hot, dry summers (May–June) and cold winters (December–January). The mean minimum and maximum temperatures of summer are 26.4 and 44.1 °C, respectively, while the mean minimum and maximum temperatures of winter are 5.6 and 23.7 °C, respectively. The annual average maximum and minimum temperatures are 40.5 and 6.5 °C, respectively. The average annual rainfall is about 760 mm, while 80% of rainfall is received from July–September. Rice straw was collected, air dried, and chopped into pieces before using it in the experiment. The detailed properties of soil and rice straw are described in the Table 5.

**Table 5:** Characteristics of soil and rice straw.

	Properties of soil
Soil particle size distribution	Sand, silt, and clay content of ~ 67.2, 14.8, and 18.0%, respectively
Soil reaction	Neutral (pH = 8.12)
Soil organic carbon	6.30 g kg^−1^
Bulk density	1.27 Mg m^−3^
Plant available P (NaHCO_3_-P)	11.6 mg kg^−1^
Plant available K (CH_3_COONH_4_-K)	220 kg ha^−1^
Soil mineral N	267 kg ha^−1^
Si availability	48.3 mg kg^−1^
Moisture content of air-dried soil	11.65%
Dehydrogenase	7.10 µg TPF g soil^−1^ h^−1^
Alkaline phosphatase activity	80.25 µg PNP g soil^−1^ h^−1^
Cellulase activity	1.09 µg glucose g soil^−1^ h^−1^
Peroxidase activity	0.46 µg ABTS g soil^−1^ h^−1^
NH_4_Cl-P	7.39 mg kg^−1^
NaOH-P	30.79 mg kg^−1^
NH_4_F-P	25.46 mg kg^−1^
H_2_SO_4_-P	618.19 mg kg^−1^
CBD-P	114.36 mg kg^−1^
Organic P	289.21 mg kg^−1^

	Properties of rice straw
Carbon	51.76%
Nitrogen	0.65%
Phosphorus	0.17%
Potassium	1.83%
Sulphur	0.06%
Silicon	6.37%

### Experiment 1: incubation experiment and measurement of P mineralization and hysteresis

The soil was treated using various amounts of rice straw and phosphorus-solubilizing microbes. The soils (100 g, oven-dried weight) were re-equilibrated to 80% of their water-holding capacity after being pre-incubated for 5 days at 27 °C^30^. The previously incubated soils were treated with phosphorus-solubilizing microbes and varied rice straw concentrations. The soil was inoculated with liquid cultures of *Pseudomonas striata* (10^8^ CFU mL^−1^) and *Aspergillus niger* (10^8^ spores mL^−1^) obtained from the Division of Soil Science and Agricultural Chemistry, ICAR-IARI, New Delhi, India. After that, rice straw was applied to the soil with and without phosphorus-solubilizing microbes at concentrations of 3570 mg kg^−1^ (i.e. 8 Mg ha^−1^), 4465 mg kg^−1^ (i.e. 10 Mg ha^−1^), 5355 mg kg^−1^ (i.e. 12 Mg ha^−1^), and 6250 mg kg^−1^ (i.e. 14 Mg ha^−1^). Ten combinations were achieved, namely: T0 (control: no phosphorus-solubilizing microbes and no rice straw), T1 (3570 mg rice straw kg^−1^ soil), T2 (3570 mg rice straw kg^−1^ soil with phosphorus-solubilizing microbes), T3 (4465 mg rice straw kg^−1^ soil), T4 (4465 mg rice straw kg^−1^ soil with phosphorus-solubilizing microbes), T5 (5355 mg rice straw kg^−1^ soil), T6 (5355 mg rice straw kg^−1^ soil with phosphorus-solubilizing microbes), T7 (6250 mg rice straw kg^−1^ soil), T8 (6250 mg rice straw kg^−1^ soil with phosphorus-solubilizing microbes), and T9 (phosphorus-solubilizing microbes only). The treated soils were stored in incubators for 90 days in three replications, preserving moisture content at 0.033 MPa and temperatures at 25 and 35 °C. By extracting soil P with 0.5 M NaHCO_3_ (pH = 8.5) the plant-available forms of P were determined after the 2nd, 7th, 15th, 30th, 45th, 60th, and 90th days of incubation^31^. After 90 days of incubation, using the modified P fractionation scheme of Kuo^32^, different forms of inorganic soil P, including saloid-bound P, Al-bound P, Fe-bound P, reductant soluble P, and Ca-bound P, were measured. These P forms included ammonium chloride extractable P (NH_4_Cl-P), ammonium fluoride extractable P (NH_4_F-P), sodium hydroxide extractable P (NaOH-P), citrate-bicarbonate extractable P (CBD-P), and sulfuric acid extractable P (H_2_SO_4_-P). As soil P mobilisation occurs by solubilisation of inorganically bound P and mineralization of organic P, we selected the following two-pool model to study its kinetics^5^

1 $$Pt={P}_{0}\left[1-{e}^{\left(-k1\times t\right)}\right] + k2t$$

Pt denotes P that is still accessible in Eq. 1 after t days of incubation. P_0_ is mineralizable organic P. The values of k1 and k2 represent the rates of inorganic P solubilisation and organic phosphorus mineralization, respectively.

Three grams of soil from each 90-day incubated sample were obtained, together with 30 mL of CaCl_2_ solution (concentration: 0.01 M and ionic strength: 0.03) containing P, 0, 5, 10, 20, 30, 50, 70, and 100 mg L^−1^, to evaluate the effects of various hydrothermal regimes on phosphate sorption. After centrifugation and decantation, the soil was again exposed to the same CaCl_2_ solution and mixed for the same amount of time as in the sorption kinetics in order to investigate phosphate desorption kinetics. For the sorption and desorption study, P concentration was estimated. The P concentrations during adsorption and desorption were fitted to Langmuir and Freundlich isotherms.

2 $$\frac{c}{x}= \frac{1}{{S}_{m}{k}_{L}}+ \frac{c}{{S}_{m}}$$

3 $$x={k}_{F}\times {c}^{n}$$

The parameter x denoted the adsorbed anion concentration (mg kg^−1^), c the equilibrium anion concentration (mg L^−1^), k_L_ the adsorption energy parameter, and S_m_ the maximal adsorption capacity (mg kg^−1^). The adsorption constant, abbreviated k_F_, denotes the energy of adsorption on a uniform surface, whereas the parameter n has a value between 0 and 1. The difference between the centre regions underneath the sorption (As) and desorption (Ad) lines was used to determine the hysteresis index (H)^33^,

4 $$H= \frac{\left(As-Ad\right)\times 100\%}{As}$$

Si was extracted from soils using CaCl_2_ solution (concentration: 0.01 M. 1:10 ratio and shaking time 16 h) following 90 days of incubation^34^ and measured spectrophotometrically using the molybdate blue colour technique^35^.

### Experiment 2: pot experiment using efficient combinations of rice straw and phosphorus-solubilizing microbes

To evaluate the efficacy of rice straw with phosphorus-solubilizing microbes to lower P fertiliser needs while maintaining crop production, pot experiment research was undertaken. Based on the findings of the laboratory investigation, six fertiliser management strategies were chosen for the trial: M0, control; M1, rice straw at 12 Mg ha^−1^ with phosphorus-solubilizing microbes with 50% recommended dose of P; M2, rice straw at 12 Mg ha^−1^ with phosphorus-solubilizing microbes with 75% recommended dose of P; M3, rice straw at 14 Mg ha^−1^ with phosphorus-solubilizing microbes with 50% recommended dose of P; M4, rice straw at 14 Mg ha^−1^ with phosphorus-solubilizing microbes with 75% recommended dose of P; and M5, 100% recommended dose of P. In all treatments, recommended doses of nitrogen and potassium were applied. The recommended optimum doses of nitrogen, phosphorus and potassium doses were 267, 45 and 135 mg pot^−1^, respectively for the maximum yield of wheat under irrigation^8^. The nutrient elements were applied through urea, superphosphate, and muriate of potash. Wheat (cv HD 2967) was chosen as a test crop because it is cultivated in a large area immediately after harvesting rice (mid-October) in India. Wheat was sown in the first week of November and harvested in the first week of April. To collect wheat and soil samples during crown root initiation (crown root initiation), flowering (FLO), and maturity (MAT) stages of wheat, separate pots with these treatments were maintained for each stage. Three replicates were tested for each treatment in each crop growth stage. Hence, a total of 54 pots (6 treatments × 3 replications × 3 growth stages) were filled with 5 kg soils for experiment 2. After the wheat crop was harvested, rice (*O. sativa*; PB 1692) was grown in the same pots on the leftover fertility. In each pot, eight seedlings were transplanted, and after establishment, it was thinned to a population of five plants per pot consistently. Nitrogen was applied at three split doses at 22 mg kg^−1^ soil, once at transplanting of the seedlings, at the maximum tillering stage and during the booting stage of rice. Before transplanting, all treatments with the exception of the absolute control received a potassium application of 27 mg K kg^−1^ soil. The required agronomic package of practices was strictly followed. Throughout the experiment, the pots were kept continuously submerged, with a maximum water depth of 2 cm. Above-ground parts of the plants were harvested after attaining physiological maturity.

### Soil and plant sampling

Soil from the pots was collected during crown root initiation, flowering, and maturity stages of the wheat lifecycle after uprooting the whole plants from the pot. Soil samples were collected at the tiller initiation (TI), panicle initiation (PI) and maturity (MAT) stages of rice to estimate available P, Si, inorganic fraction of P, enzyme activity, and organic acid concentrations.

The concentrations of organic acids in the soil were estimated using a high-performance liquid chromatography (HPLC). The amount of inorganic P fractions were measured and organic P was quantified by the ignition method^32^. Si concentration^34^ and soil pH were also measured in the soil. Activities of enzymes, such as dehydrogenase, alkaline phosphatase, cellulase, and peroxidase were estimated in soil using tetrazolium chloride^36^, p-nitro phenyl-phosphate^37^, carboxymethyl cellulose^38^, and ABTS^39^ as substrates, respectively. At each stage of the sampling process, whole plant samples were carefully taken from each pot. Roots from the plant samples were then cleaned twice–once with tap water and once with distilled water—and then root length and volume were measured using a root scanner. Individual masses of straw and grain shoot were measured at the maturity stage. A Wiley mill was used to grind the oven-dried plant samples, which were then digested with di-acid (HNO_3_:HClO_4_) at 9:4 ratio and spectrophotometrically measured for total P content using the vanadomolybdophosphate yellow colour technique^40^.

### Statistical analyses

All the data were analysed using the Analysis of Variance (ANOVA) technique, and the Tukey’s Honestly Significant Difference (HSD) test was employed to distinguish between significantly different mean values for every variable. We developed a path model (also known as a structural equation model) for the wheat-rice using various latent variables to evaluate the direct and indirect effects of several factors, including soil chemical environment (pH and Si), microbial environment (activities of dehydrogenase, alkaline phosphatase, cellulase, and peroxidase), and plant-microbial factor (organic acids) on P mobilisation in the rhizosphere of wheat and rice. Strong model fits were indicated by high GFI (> 0.9) and low RMSEA (< 0.08). The magnitude of the arrows determines the overall influence of latent variables.

### Permission statement

Appropriate permission for collection and use of rice straw was obtained from the competent authority.

### Laboratory analysis statement

All methods and analysis were carried out in accordance with relevant guidelines and regulations.

## Data Availability

All data generated or analysed during this study are included in this article.
